# Neuromuscular impairment at different stages of human sarcopenia

**DOI:** 10.1002/jcsm.13531

**Published:** 2024-09-05

**Authors:** Fabio Sarto, Martino V. Franchi, Jamie S. McPhee, Daniel W. Stashuk, Matteo Paganini, Elena Monti, Maira Rossi, Giuseppe Sirago, Sandra Zampieri, Evgeniia S. Motanova, Giacomo Valli, Tatiana Moro, Antonio Paoli, Roberto Bottinelli, Maria A. Pellegrino, Giuseppe De Vito, Helen M. Blau, Marco V. Narici

**Affiliations:** ^1^ Department of Biomedical Sciences University of Padova Padova Italy; ^2^ CIR‐MYO Myology Centre University of Padova Padova Italy; ^3^ Department of Sport and Exercise Sciences Manchester Metropolitan University Institute of Sport Manchester UK; ^4^ Department of Systems Design Engineering University of Waterloo Waterloo ON Canada; ^5^ Baxter Laboratory for Stem Cell Biology, Department of Microbiology and Immunology Stanford University School of Medicine Stanford CA USA; ^6^ Institute of Physiology, Department of Molecular Medicine University of Pavia Pavia Italy; ^7^ Institute of Sport Sciences and Department of Biomedical Sciences University of Lausanne Lausanne Switzerland; ^8^ Department of Surgery, Oncology, and Gastroenterology University of Padova Padova Italy; ^9^ IRCCS Mondino Foundation Pavia Italy

**Keywords:** electromyography, fibre denervation, motoneuron, motor units, muscle atrophy, neuromuscular junction

## Abstract

**Background:**

Degeneration of the motoneuron and neuromuscular junction (NMJ) and loss of motor units (MUs) contribute to age‐related muscle wasting and weakness associated with sarcopenia. However, these features have not been comprehensively investigated in humans. This study aimed to compare neuromuscular system integrity and function at different stages of sarcopenia, with a particular focus on NMJ stability and MU properties.

**Methods:**

We recruited 42 young individuals (Y) (aged 25.98 ± 4.6 years; 57% females) and 88 older individuals (aged 75.9 ± 4.7 years; 55% females). The older group underwent a sarcopenia screening according to the revised guidelines of the European Working Group on Sarcopenia in Older People 2. In all groups, knee extensor muscle force was evaluated by isometric dynamometry, muscle morphology by ultrasound and MU potential properties by intramuscular electromyography (iEMG). MU number estimate (iMUNE) and blood samples were obtained. Muscle biopsies were collected in a subgroup of 16 Y and 52 older participants.

**Results:**

Thirty‐nine older individuals were non‐sarcopenic (NS), 31 pre‐sarcopenic (PS) and 18 sarcopenic (S). A gradual decrease in quadriceps force, cross‐sectional area and appendicular lean mass was observed across the different stages of sarcopenia (for all *P* < 0.0001). Handgrip force and the Short Physical Performance Battery score also showed a diminishing trend. iEMG analyses revealed elevated near fibre segment jitter in NS, PS and S compared with Y (Y vs. NS and S: *P* < 0.0001; Y vs. PS: *P* = 0.0169), suggestive of age‐related impaired NMJ transmission. Increased C‐terminal agrin fragment (*P* < 0.0001) and altered caveolin 3 protein expression were consistent with age‐related NMJ instability in all the older groups. The iMUNE was lower in all older groups (*P* < 0.0001), confirming age‐related loss of MUs. An age‐related increase in MU potential complexity was also observed. These observations were accompanied by increased muscle denervation and axonal damage, evinced by the increase in neural cell adhesion molecule‐positive fibres (Y vs. NS: *P* < 0.0001; Y vs. S: *P* = 0.02) and the increase in serum concentration of neurofilament light chain (*P* < 0.0001), respectively. Notably, most of these MU and NMJ parameters did not differ when comparing older individuals with or without sarcopenia.

**Conclusions:**

Alterations in MU properties, axonal damage, an altered innervation profile and NMJ instability are prominent features of the ageing of the neuromuscular system. These neuromuscular alterations are accompanied by muscle wasting and weakness; however, they appear to precede clinically diagnosed sarcopenia, as they are already detectable in older NS individuals.

## Introduction

Sarcopenia, the progressive loss of skeletal muscle mass and function with ageing,^1^, ^2^ is recognized as a clinical disorder that affects individuals' quality of life and leads to an increased risk of falls, functional decline, frailty and mortality.^2^ Sarcopenia is increasingly common in older populations (≥60 years of age), with ranges between 10% and 27% depending on the classification employed,^3^ and represents an enormous burden on the healthcare system.^2^ The pathophysiology of sarcopenia is complex, multifactorial and not yet fully elucidated. Evidence suggests that this disorder has a key neurogenic component.^1^, ^4^ A reduced motor unit (MU) number with ageing due to motoneuron death is a consistent finding in both anatomical and electrophysiological studies.^5^ This process is accompanied by recurring cycles of denervation–reinnervation inducing remodelling of the MU, fibre atrophy and fibre type grouping.^4^ Neuromuscular junctions (NMJs), the specialized synapses connecting motoneuron axonal branches with skeletal muscle fibres,^6^ also exhibit extensive remodelling with ageing. NMJ morphological changes include increased dimensions and complexity of nerve terminal branching, a decreased number of acetylcholine (ACh)‐containing vesicles, active zones, ACh receptors and junctional folds, NMJ denervation and increased endplate fragmentation.^4^, ^6^, ^7^ In addition to this anatomical remodelling, ageing triggers adaptations to NMJ physiological function, leading to a decreased safety factor and an increased incidence of neurotransmission failure.^8^ While these findings imply that degeneration of motoneurons and NMJs and MU loss may underlie the muscle wasting and weakness observed in sarcopenia, these observations derive mostly from animal models. Furthermore, the evidence available from human studies is generally limited to comparisons between young and older individuals, and a diagnosis of sarcopenia using official guidelines is rarely used. Some of the previous studies on clinically diagnosed sarcopenia focused on circulating biomarkers of neurodegeneration,^9^, ^10^ electromyographic techniques to investigate the integrity of the neuromuscular system^11^, ^12^ or immunohistochemistry to assess muscle fibre morphology.^13^ A comprehensive evaluation of the different components of the MU combining these different approaches in the context of human sarcopenia is currently lacking.

In this study, we aimed to investigate muscle morphological, functional, electrophysiological and molecular alterations at different stages of primary sarcopenia, in which no other discernible cause was apparent,^2^ in older humans. In particular, we focused on NMJ alterations and MU potential (MUP) properties. We hypothesized that a gradual impairment of the neuromuscular system would be a hallmark of the progression of sarcopenia.

## Material and methods

### Participants

Forty‐two healthy young individuals (Y) (57% females) and 88 older individuals (55% females) volunteered for this study. Inclusion criteria were 18–35 years of age for Y and >70 years for older adults. Exclusion criteria for both groups were high levels of physical activity (>4 sessions/week) and, as we focused on primary sarcopenia, the presence of potentially confounding neurological disorders, diabetes with complications, stroke, late‐stage cancer, chronic kidney disease (stages IV and V), severe liver insufficiency, severe cardiac disorder and serious lower limb musculoskeletal injuries in the last 12 months and an implantable cardioverter‐defibrillator. This study was carried out following the guidelines of the most recent version of the Declaration of Helsinki and received approval from the Ethics Committee of the Department of Biomedical Sciences, University of Padova (Italy) (n. HEC‐DSB/03‐20). Before data collection, volunteers were informed about all the experimental procedures and provided written consent.

### Experimental protocol

In this cross‐sectional study, volunteers were asked to visit the laboratory two to three times. On the first visit (only for older adults), participants underwent a full sarcopenia screening, including the handgrip test, the Short Physical Performance Battery (SPPB) and a whole‐body dual‐energy X‐ray absorptiometry (DEXA) scan. On the second visit (for both groups), the Global Physical Activity Questionnaire (GPAQ), a blood sample, ultrasound scans, in vivo muscle function assessment and intramuscular electromyography (iEMG) recordings were collected. In a subgroup of participants (Y: *n* = 16; older adults: *n* = 52), vastus lateralis muscle biopsies were obtained during a third visit. Visits were separated by 2–7 days. Participants were asked to refrain from any vigorous form of exercise during the 24 h preceding each visit.

### Sarcopenia screening and classification

Handgrip strength was evaluated following the recommendations of the European Working Group on Sarcopenia in Older People 2 (EWGSOP2)^2^ using a hand‐held dynamometer (Jamar Plus+, JLW Instruments, Chicago, IL, USA). Further details can be found in *Data S1*.

Physical performance was assessed employing the SPPB, which includes three subtests for the evaluation of static balance, gait speed over a 4‐m walk and the ability to stand repetitively five times from a chair as fast as possible (chair‐to‐stand test [CST]). Further details can be found in *Data S1*.

Body composition was evaluated by DEXA (Hologic Horizon™ QDR RSeries, Bedford, MA, USA). Appendicular lean mass (ALM) was computed as the sum of arms and legs lean mass.

#### Sarcopenia classification

Older participants were classified according to the guidelines of the EWGSOP2,^2^ with the modified thresholds for handgrip strength recently proposed by Westbury and colleagues.^14^ Accordingly, the coexistence of reduced muscle strength and decreased lean mass was considered indicative of sarcopenia.^2^ Because we were also interested in early neuromuscular alterations, the pre‐sarcopenic individuals were identified based on either reduced muscle strength or decreased lean mass, as in previous studies.^9^ For males, low muscle strength was defined as handgrip strength < 35.5 kg and/or CTS time > 15 s, while low lean mass was defined as ALM < 20 kg and/or ALM/h^2^ < 7.0 kg/m^2^. For females, low muscle strength was defined as handgrip strength < 20 kg and/or CTS time > 15 s, while low lean mass was defined as ALM < 15 kg and/or ALM/h^2^ < 5.5 kg/m^2^.

### In vivo muscle morphology and function

#### Muscle size measurements

Quadriceps femoris cross‐sectional area (CSA) was assessed at three different muscle regions (30%, 50% and 70% of femur length) using panoramic ultrasound imaging (MyLab70, Esaote, Genoa, Italy).^15^ CSA was determined by tracing the muscle contours using ImageJ software (1.52v; National Institutes of Health, Bethesda, MD, USA). The CSA_mean_ of the quadriceps and vastus lateralis was computed by averaging the CSA values at the three different muscle regions. Vastus lateralis muscle architecture was evaluated by B‐mode longitudinal ultrasound scans at 50% of femur length. Fascicle length (Lf) and pennation angle (PA) were obtained using ImageJ. Further details regarding image acquisition and analysis can be found in *Data S1*.

#### Quadriceps force, rapid force production and activation capacity

Quadriceps force was evaluated by isometric dynamometry at 90° of knee flexion. Three trials were recorded, and the highest value reached was considered the maximum voluntary contraction (MVC). This was then normalized to quadriceps CSA_mean_ to evaluate the specific tension. The time required to reach 63% of MVC (TTP63%) was used as an index of rapid force production. Finally, activation capacity was evaluated using the interpolated twitch technique. For further information regarding these procedures, we refer the reader to Monti et al.^16^ and *Data S1*.

### Intramuscular electromyography

The vastus lateralis central motor point was detected with low‐intensity percutaneous electrical stimulations, as previously described.^15^ iEMG recordings were obtained during isometric contractions at 10% and 25% MVC using a concentric needle electrode of 25 or 37 mm, depending on muscle and subcutaneous fat thickness, inserted at the motor point (S53155 or S53155, Teca Elite, Natus Medical Inc., Middleton, WI, USA). Twelve 20‐s contractions were collected (six at 10% MVC and six at 25% MVC), adjusting the needle position for each pair of contractions. An experienced operator employed DQEMG software to extract MUP trains and carry out all the analyses.^17^ The markers, relative to MUP onset, end, positive peak and negative peak, were manually adjusted, where appropriate. The following parameters were evaluated^15^, ^18^: (i) MUP area; (ii) MUP duration; (iii) number of turns (i.e., a change in MUP waveform of at least 20 μV), indicative of the complexity of the MUP; and (iv) MU mean firing rate.

Near fibre MUP (NF MUP) trains were obtained after bandpass filtering MUPs using a second‐order low‐pass differentiator^19^ and were visually inspected, manually excluding NF MUPs containing contaminating activity generated by other MUs. The NF MUP properties analysed were NF MUP duration and NF count, a measure of fibre density contributing to the NF MUP trains. NMJ transmission instability was evaluated using NF MUP segment jitter, which assesses temporal variability (i.e., mean absolute consecutive temporal differences) in consecutive NF MUPs and is considered correlated with the NMJ safety factor.^20^ An MU number estimate (iMUNE) was also computed as the ratio between the mean MUP area and the vastus lateralis CSA_mean_.^21^ For further details regarding these procedures, we refer the reader to Sarto et al.^15^ and *Data S1*.

### Blood sampling and circulating biomarkers assessment

Blood samples were collected from the medial cubital vein, and serum was obtained by centrifugation at 3000 rpm for 10 min. Circulating biomarkers involved in neuromuscular degeneration were measured, including C‐terminal agrin fragment (CAF), neurofilament light chain and neurotrophins brain‐derived neurotrophic factor (BDNF) and neurotrophin‐4 (NT‐4). In addition, we evaluated inflammation levels by dosing interleukin‐6 (IL‐6). All the analyses were performed using enzyme‐linked immunosorbent assay (ELISA) kits, apart from the neurofilament light chain concentration that was obtained employing the single‐molecule array (SIMOA) technology. Further details can be found in *Data S1*.

### Muscle biopsy

In a subgroup of participants (Y: *n* = 16; older adults: *n* = 52), a vastus lateralis muscle biopsy was obtained with a Weil‐Blakesley conchotome (Gebrüder Zepf Medizintechnik GmbH & Co. KG, Dürbheim, Germany) under local anaesthesia (5 mL of lidocaine at 2%). Each sample was collected ~2 cm proximally from the motor point. The sample was divided into a part for western blot analysis and another one for immunofluorescence analysis. We assessed the protein levels of selected downstream components of the agrin pathway, the expression of neural cell adhesion molecule (NCAM)‐positive fibres and the variability of muscle fibre diameters. Further details can be found in *Data S1*.

### Statistical analysis

A two‐way analysis of variance (ANOVA) (Group × Sex) with Holm post hoc was performed to determine differences between different stages of sarcopenia. Variables not normally distributed were analysed employing the Kruskal–Wallis test with Dunn's multiple comparisons tests. As multiple MUs were sampled from each participant, iEMG data were analysed using generalized linear mixed models (fixed effects: group and sex; cluster variable: subject). Statistical significance was accepted at *P* ≤ 0.05. For a full description of the statistical analyses, we refer the reader to *Data S1*.

## Results

According to the sarcopenia screening, the older individuals (age range: 70–91 years) were categorized as non‐sarcopenic (NS; *n* = 39), pre‐sarcopenic (PS; *n* = 31) or sarcopenic (S; *n* = 18). Participants' characteristics for each group are reported in *Table* *1*. All participants were included in the analysis, unless otherwise reported. Figure captions report whether any data point was not collected or removed due to technical issues. Statistical tests showed that, for most of the parameters considered, no interaction between group and sex existed. Thus, males and females are pooled together in the main text results. Full results regarding the sex effect can be found in *Data S1*.

**Table 1:** Participants' characteristics, body composition and components of the Short Physical Performance Battery (SPPB)

	Group				*P*‐value (group)						*P*‐value (sex)
	Young (Y)	Non‐sarcopenic (NS)	Pre‐sarcopenic (PS)	Sarcopenic (S)	Y–NS	Y–PS	Y–S	NS–PS	NS–S	PS–S	M–F
*N* TOT	42	39	31	18	/	/	/	/	/	/	/
*N* F/M	24/18	17/22	19/12	12/6	/	/	/	/	/	/	/
Age (years)	25.98 (4.52)	74.8 (3.76)	74.97 (4.21)	79.83 (5.14)	**<0.0001**	**<0.0001**	**<0.0001**	0.865	**0.001**	**0.001**	0.500
Height (m)	1.71 (0.08)	1.69 (0.08)	1.60 (0.09)	1.55 (0.08)	**0.019**	**<0.0001**	**<0.0001**	**0.001**	**<0.0001**	**0.019**	**<0.0001**
Weight (kg)	66.1 (9.85)	78.66 (12.6)	67.98 (11.13)	65.11 (8.96)	**<0.0001**	0.192	0.640	0.017	**0.0001**	0.192	**<0.0001**
GPAQ score (MET‐min/week)	3330 (2100)	1526 (1200)	1370 (1200)	1517 (1440)	**<0.0001**	**<0.0001**	**<0.0001**	0.986	0.976	0.882	0.415
Body fat (%)	/	35.31 (7.44)	37.31 (6.82)	37.01 (8.63)	/	/	/	0.999	0.999	0.999	**<0.0001**
ALM/h^2^ (kg/m^2^)	/	7.34 (1.00)	6.65 (1.04)	6.41 (0.65)	/	/	/	**0.029**	**0.009**	0.999	**<0.0001**
Balance score SPPB	/	3.61 (0.78)	3.62 (0.76)	3.11 (1.08)	/	/	/	0.991	0.135	0.189	0.935
Gait speed (m/s)	/	1.12 (0.17)	1.05 (0.19)	1.06 (0.21)	/	/	/	0.387	0.744	0.744	0.249
CST time (s)	/	10.46 (2.19)	11.06 (2.56)	12.71 (3.71)	/	/	/	0.296	**0.048**	0.392	0.652

*Note*: Data are reported as mean and standard deviation. Bold *P*‐values indicate statistical significant differences.

Abbreviations: ALM, appendicular lean mass; CST, chair‐to‐stand test; F, female; GPAQ, Global Physical Activity Questionnaire; M, male; MET, metabolic equivalent; TOT, total.

### In vivo muscle function and physical performance

**Figure 1:**
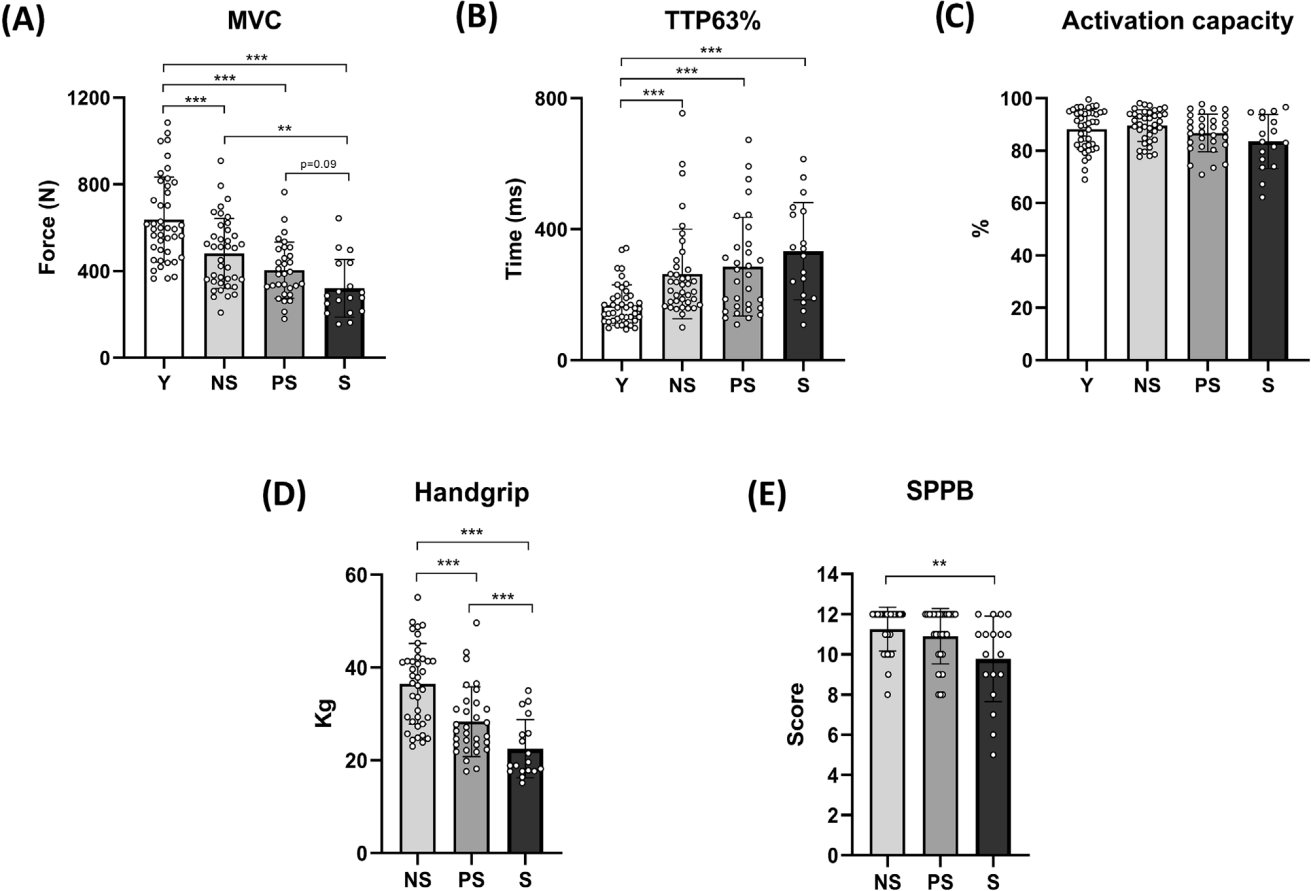
Muscle function and physical performance parameters across different stages of human sarcopenia. Statistical analysis was performed using two‐way analyses of variance (A–D) and the Kruskal–Wallis test (E). Results are shown as the mean and standard deviation. Knee extensor maximum voluntary contraction (MVC) (A); time needed to reach 63% of the MVC (TTP63%) (B); activation capacity (C); handgrip strength (D); and Short Physical Performance Battery (SPPB) score (E). Three missing values (one NS, one PS and one S) and one outlier excluded (Y; robust regression and outlier removal method with Q = 2%) for activation capacity. NS, non‐sarcopenic; PS, pre‐sarcopenic; S, sarcopenic; Y, young individuals. **P < 0.01; ***P < 0.001.

We characterized the functional impairment across sarcopenia stages (*Table* *1* and *Figure* *1*). Handgrip strength (*P* < 0.0001; ηp^2^ = 0.49) and MVC (*P* < 0.0001; ηp^2^ = 0.47) were increasingly lower from NS to S. Differences between old groups disappeared when these values were normalized for arm lean mass and quadriceps CSA_mean_, respectively (*Figure* *S1*). Activation capacity, an indicator of the ability to recruit MUs under maximal effort, did not differ across groups. The older groups showed slower rapid force production capacity (*P* < 0.0001; ηp^2^ = 0.2), as suggested by increased TTP63%, but no differences were observed when comparing NS, PS and S. NS presented a superior performance in the overall SPPB score (*P* = 0.009) and the CTS time (*P* = 0.048) compared with S. Balance score and gait speed did not differ among groups.

### In vivo muscle morphology

**Figure 2:**
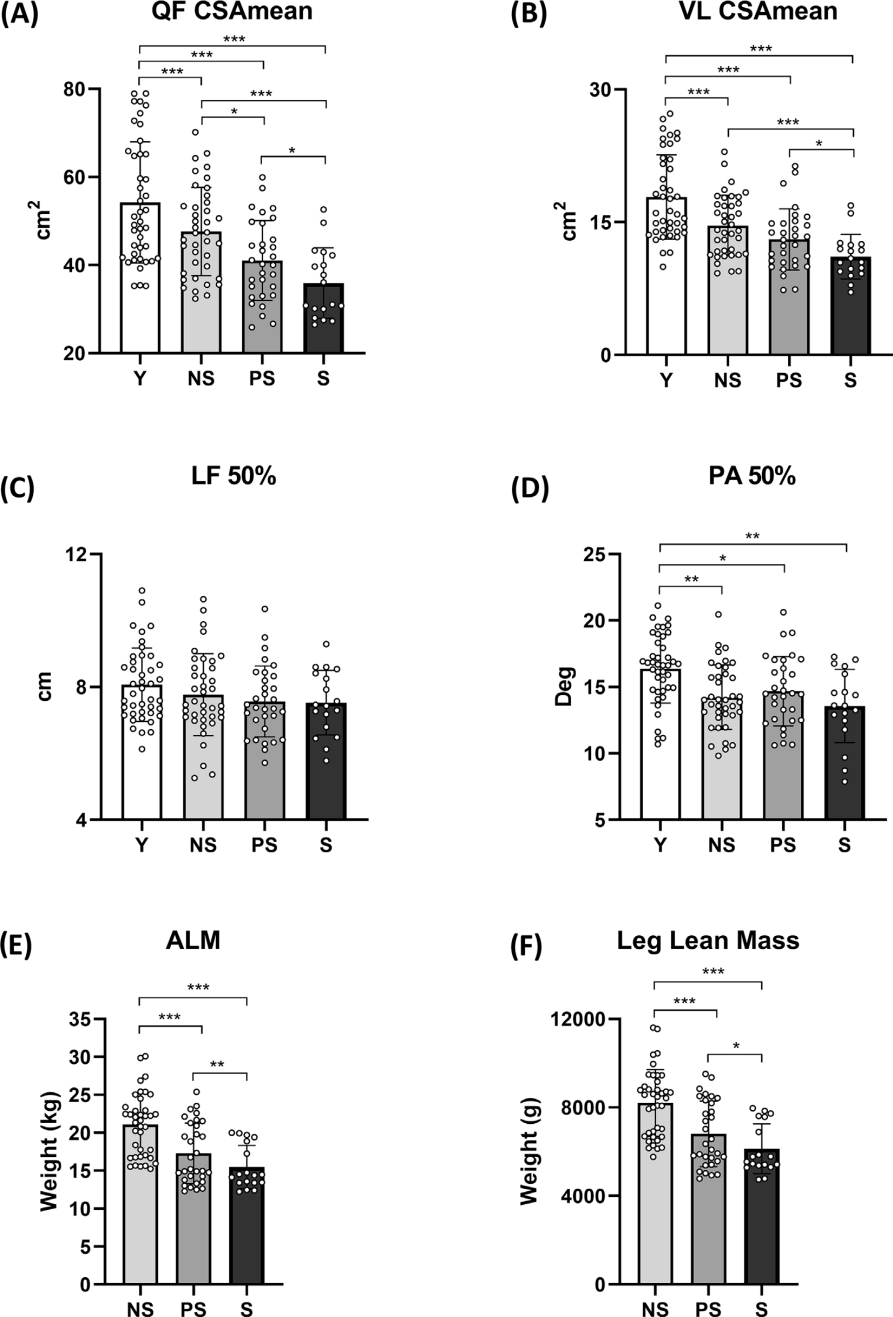
Muscle morphology parameters and lean mass quantification across different stages of human sarcopenia. Statistical analysis was performed using two‐way analyses of variance. Results are shown as the mean and standard deviation. Quadriceps femoris (QF) cross‐sectional area (CSA_mean_): mean of the values at 30%, 50% and 70% of femur length (A); vastus lateralis (VL) CSA_mean_ (B); VL fascicle length (Lf) at 50% femur length (C); VL pennation angle (PA) at 50% femur length (D); appendicular lean mass (ALM) (E); and leg lean mass (F). NS, non‐sarcopenic; PS, pre‐sarcopenic; S, sarcopenic; Y, young individuals. *P < 0.05; **P < 0.01; ***P < 0.001.

We assessed the differences in lean mass and muscle morphology by DEXA and ultrasound, respectively (*Table* *1* and *Figure* *2*). ALM (*P* < 0.0001; ηp^2^ = 0.44), ALM/h^2^ (*P* < 0.0046; ηp^2^ = 0.44) and leg lean mass (*P* < 0.0001; ηp^2^ = 0.39) showed a diminishing trend across the different stages of sarcopenia. Muscle atrophy was also evident in the gradual decreases in quadriceps CSA_mean_ (*P* < 0.0001; ηp^2^ = 0.48) and vastus lateralis CSA_mean_ (*P* < 0.0001; ηp^2^ = 0.45) with sarcopenia progression. Lf did not differ among groups; PA was lower in older groups compared with Y (*P* = 0.0002; ηp^2^ = 0.44), confirming age‐related alterations in muscle architecture.

### Motor unit number estimate, motor unit potential properties and neuromuscular junction transmission in vivo

**Figure 3:**
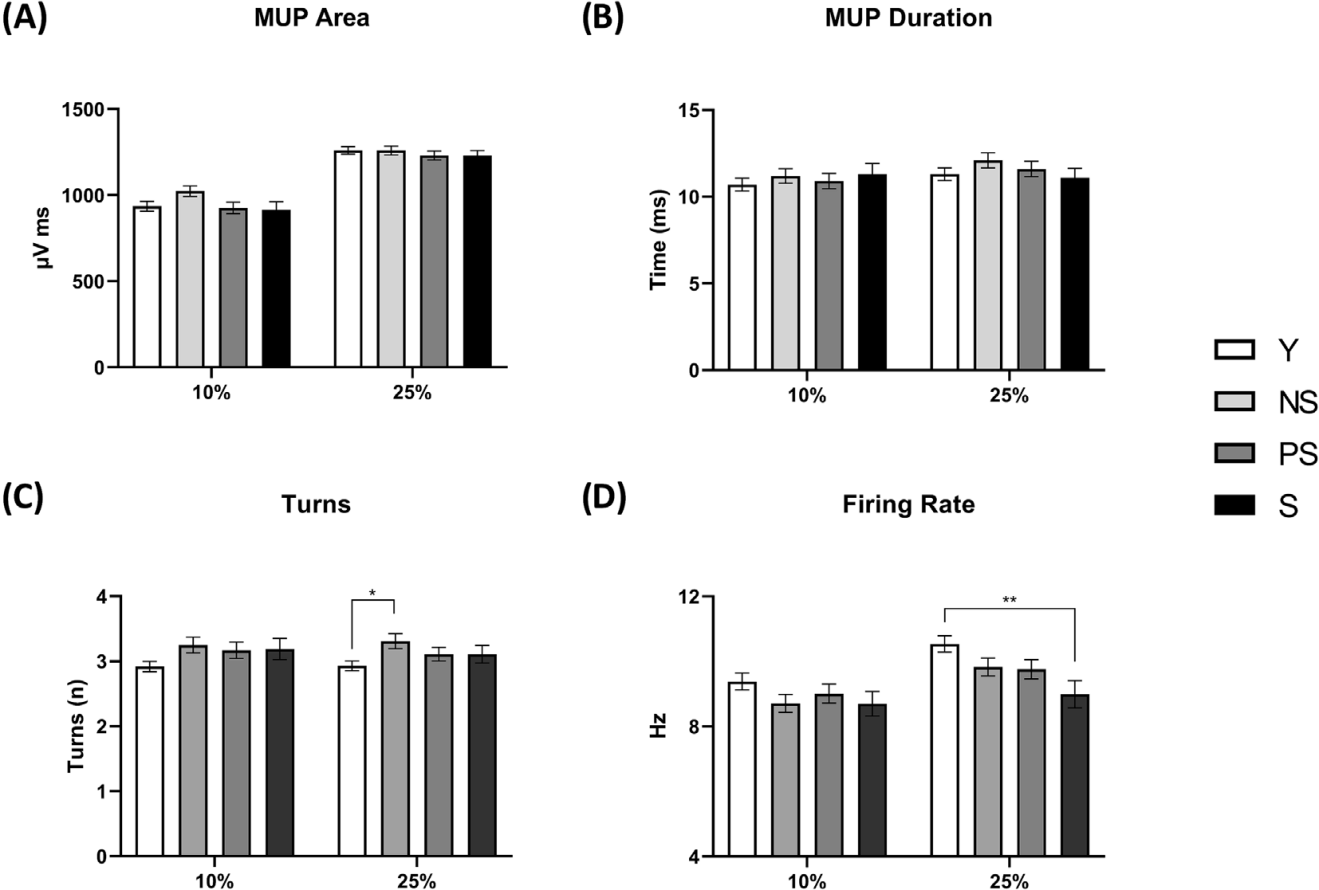
Motor unit potential (MUP) parameters across different stages of human sarcopenia. Statistical analysis was performed using generalized linear mixed models. Results are shown as the estimated marginal mean and standard error. MUP area (A); MUP duration (B); MUP turns (C); and MU mean firing rate (D). NS, non‐sarcopenic; PS, pre‐sarcopenic; S, sarcopenic; Y, young individuals. *P < 0.05; **P < 0.01.

**Figure 4:**
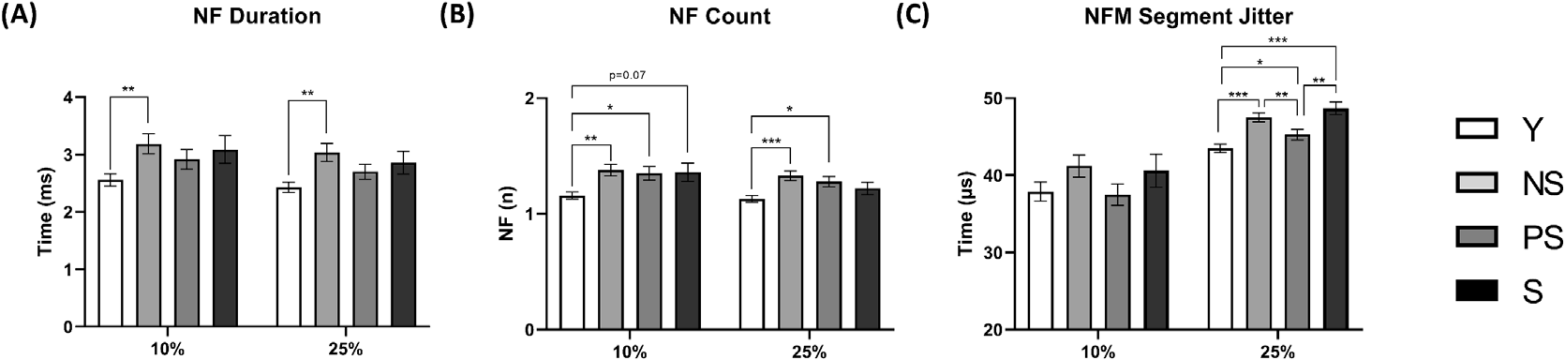
Near fibre (NF) electromyography outcomes across different stages of human sarcopenia. Statistical analysis was performed using generalized linear mixed models. Results are shown as the estimated marginal mean and standard error. NF motor unit potential (MUP) duration (A); NF count (B); and NF MUP (NFM) segment jitter (C). NS, non‐sarcopenic; PS, pre‐sarcopenic; S, sarcopenic; Y, young individuals. *P < 0.05; **P < 0.01; ***P < 0.001.

We tested whether alterations in muscle morphology and function with sarcopenia were accompanied by MU loss (*Figure* *6*) and changes in MUP properties and NMJ transmission (*Figures* *3* and *4*). The iMUNE was lower in all the older groups compared with Y (*P* < 0.0001; ηp^2^ = 0.16). The MU mean firing rate was lower in S compared with Y at 25% MVC (*P* = 0.039). MUP area and duration showed no effect of group for both contraction intensities. MUP complexity, evaluated by the number of turns, was elevated in NS compared with Y at 25% MVC (*P* = 0.0278). Similar behaviour was observed for NF MUP duration (*P* = 0.003), while NF count was increased in NS and PS compared with Y (10% MVC: Y vs. NS = 0.0045; Y vs. PS = 0.0276; 25% MVC: Y vs. NS = 0.0004; Y vs. PS = 0.0245). Age‐related NMJ transmission impairment, evaluated using the NF MUP segment jitter, was elevated in older groups with respect to Y at 25% MVC (Y vs. NS and S: *P* < 0.0001; Y vs. PS: *P* = 0.0169). Differences were also detected when comparing PS with NS (*P* = 0.008) and S (*P* = 0.0072). Additional information on the iEMG data analysis is shown in *Table* *S1*.

### Circulating biomarkers of neurodegeneration

**Figure 5:**
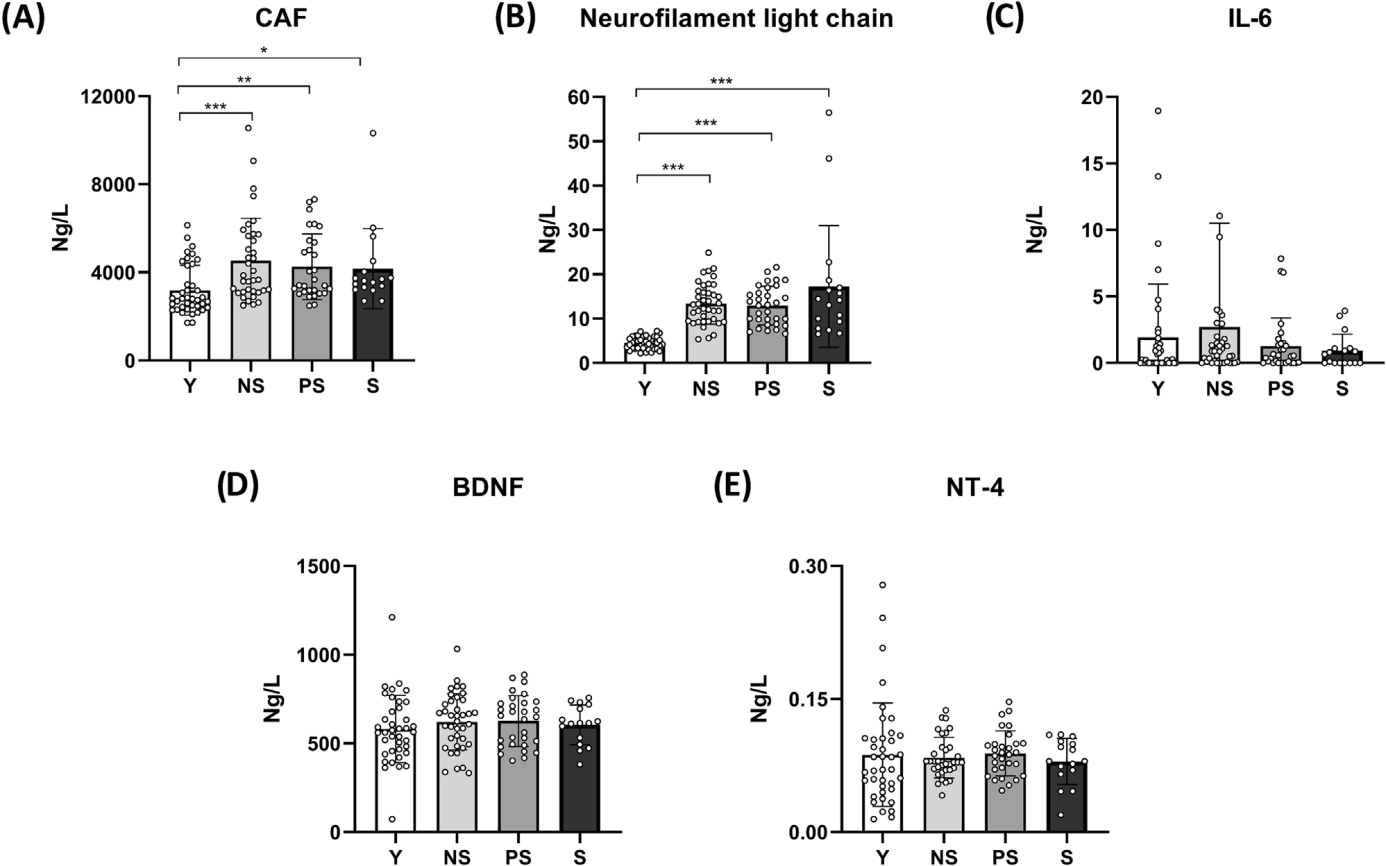
Circulating biomarker parameters across different stages of human sarcopenia. Statistical analysis was performed using Kruskal–Wallis tests (A–C) and two‐way analyses of variance (D, E). Results are shown as the mean and standard deviation. Blood concentration of C‐terminal agrin fragment (CAF) (A); neurofilament light chain (B); interleukin‐6 (IL‐6) (C); brain‐derived neurotrophic factor (BDNF) (D); and neurotrophin‐4 (NT‐4) (E). For all parameters, data missing for three Y, one NS and one S. Additional missing values are one NS for CAF, one S for BDNF and four NS and one S for NT‐4. One value is out of scale for IL‐6. NS, non‐sarcopenic; PS, pre‐sarcopenic; S, sarcopenic; Y, young individuals. *P < 0.05; **P < 0.01; ***P < 0.001.

Considering the observed changes in muscle electrophysiological properties, we assessed circulating biomarkers of neurodegeneration (*Figure* *5*). The cleavage of agrin induced by the enzyme neurotrypsin leads to the release of the soluble CAF into the bloodstream, with higher concentrations of the latter considered indicative of NMJ molecular instability.^22^ An age‐related increase in CAF concentration (*P* < 0.0001; ε^2^ = 0.178) was observed with no differences among older groups. Neurofilament light chain concentration, a biomarker of axonal damage,^9^ was significantly elevated in older groups compared with Y (*P* < 0.0001; ε^2^ = 0.621). No differences were observed when comparing the four groups for circulating neurotrophins (BDNF and NT‐4) and systemic inflammation (IL‐6).

### Muscle biomarkers of neuromuscular junction molecular instability and denervation

**Figure 6:**
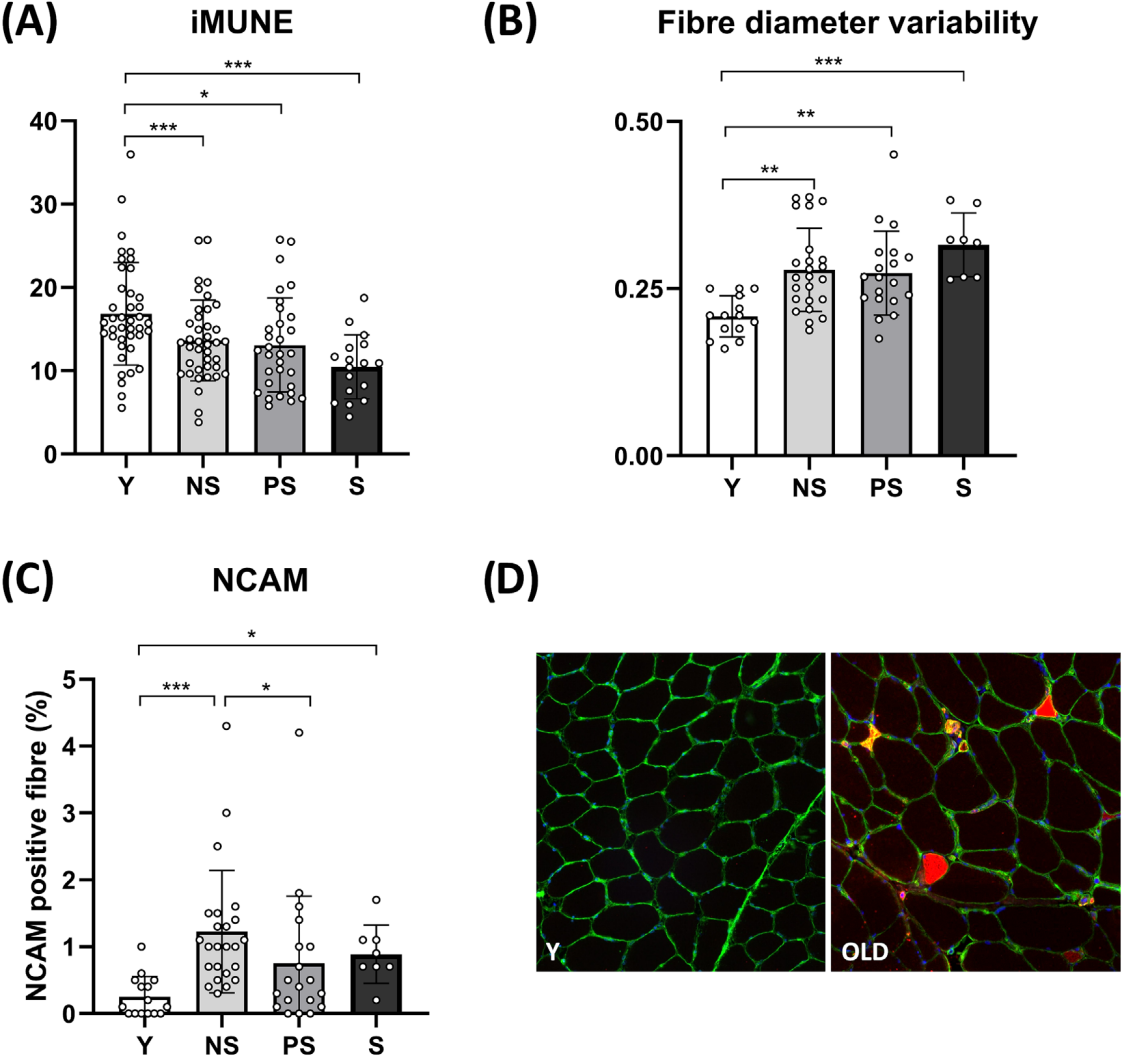
Motor unit loss and immunohistochemistry outcomes across different stages of human sarcopenia. Statistical analysis was performed using two‐way analyses of variance (A) and Kruskal–Wallis tests (B, C). Results are shown as the mean and standard deviation. Motor unit number estimate (iMUNE) (A); fibre diameter variability, expressed as coefficient of variation (B); neural cell adhesion molecule (NCAM)‐positive fibre percentage (C); and representative images of young (left panel) and aged (right panel) skeletal muscle fibres cross‐sectional area (stained by laminin in green) and NCAM+ fibres (in red) (D). Two missing values for NCAM and fibre diameter variability (one Y and one PS). Two outlier excluded for iMUNE (one Y and one S; robust regression and outlier removal method with Q = 2%). NS, non‐sarcopenic; PS, pre‐sarcopenic; S, sarcopenic; Y, young individuals. *P < 0.05; **P < 0.01; ***P < 0.001.

**Figure 7:**
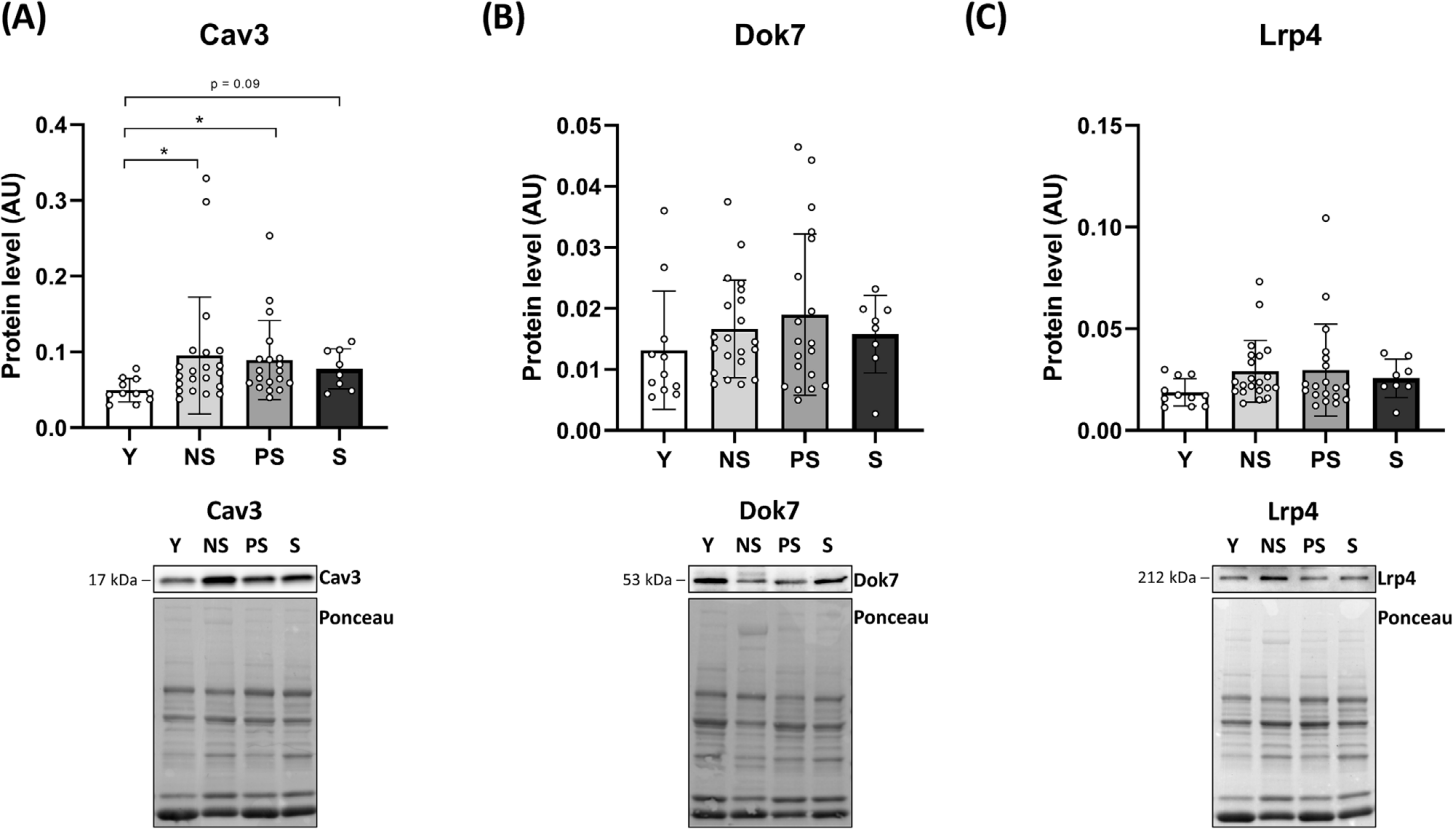
Protein levels of some downstream elements of the agrin pathway across different stages of human sarcopenia and representative western blots. Statistical analysis was performed using Kruskal–Wallis tests. Results are shown as the mean and standard deviation. Caveolin 3 (Cav3) (A); docking protein 7 (Dok7) (B); and low‐density lipoprotein receptor‐related protein 4 (Lrp4) (C). The intensity of immunostained bands was normalized to the total protein amount measured from the same membrane stained with Ponceau S staining. Data presented for 11 Y, 21 NS, 19 PS and 8 S. NS, non‐sarcopenic; PS, pre‐sarcopenic; S, sarcopenic; Y, young individuals. *P < 0.05.

We evaluated skeletal muscle biopsies to gain insights into the potential mechanisms driving NMJ degeneration and MUP alterations in ageing (*Figures* *6*, *7*, *S2* and *S3*). First, we assessed the principal downstream proteins of the agrin pathway and the predominant molecular mechanisms regulating ACh receptor clustering and NMJ endplate fragmentation.^6^ We did not observe differences between groups in lipoprotein receptor‐related protein 4 (Lrp4), total muscle‐specific kinase (MuSK), phosphorylated MuSK_(Tyr755)_ and docking protein 7 (Dok7) protein levels. Similarly, we did not find differences in protein levels of the ACh receptor δ, γ and ε subunits (*Figure* *S2*). However, caveolin 3 (Cav3) was increased in NS (*P* = 0.0337) and PS (*P* = 0.0145) compared with Y, with a trend also for S (*P* = 0.09). Cav3 is a structural protein component of caveolae in muscle fibres involved in agrin‐induced ACh receptor clustering through MuSK phosphorylation/activation.^23^ In addition, an increased percentage of NCAM‐positive fibres was observed in Y versus NS (*P* < 0.0001; 3.8‐fold) and Y versus S (*P* = 0.02; 2.5‐fold), highlighting remodelling of the innervation pattern with ageing.^24^ NS exhibited higher NCAM+ fibres than PS (*P* = 0.0477; +60%). Finally, the variability of muscle fibre diameter was greater in all old groups compared with Y (*P* < 0.0001; ε^2^ = 0.326).

## Discussion

This study represents a comprehensive description of the neuromuscular alterations occurring at different stages of primary sarcopenia in older humans. The main findings are that (i) loss of MUs, NMJ instability, NMJ transmission impairment, myofibre denervation and axonal damage are prominent features of ageing of the neuromuscular system in older individuals and (ii) these neuromuscular alterations are accompanied by loss of muscle strength and size, and they were present in all the older groups, irrespective of sarcopenia status (NS, PS or S), as clinically diagnosed.

### Alterations in neuromuscular junction transmission and stability

As expected, S displayed an overall lower level of muscle function and physical performance in comparison to PS and NS. This refers not only to the parameters included in the sarcopenia definition (handgrip strength and CTS time) but also to the knee extensor MVC. Males and females were similarly affected. Alteration in NMJ function is increasingly recognized as a driver of muscle weakness in the context of ageing and sarcopenia.^8^ Our findings support this view, demonstrating an increased NMJ segment jitter indicative of impaired NMJ transmission^18^, ^20^ in all old cohorts compared with Y. However, S did not exhibit a greater impairment compared with NS. While there is convincing evidence showing age‐related alterations in jitter values both in rodents^25^ and in humans,^26^ data in older humans clinically diagnosed with sarcopenia are rare. Only one published study was performed on this topic. This study is based on a small sample (total *n* = 19) of pre‐sarcopenic, sarcopenic and severely sarcopenic older individuals and found elevated NF segment jitter in individuals with severe sarcopenia compared with those with pre‐sarcopenia.^11^ Importantly, the present iEMG results of altered NMJ function are in line with our molecular data on NMJ integrity. Indeed, we found an age‐related increase in serum CAF concentration, a well‐established biomarker of NMJ instability,^22^ common to all the older groups. Ageing per se is known to result in an increased CAF concentration, although this biomarker has generally been reported to be further elevated in sarcopenic compared with non‐sarcopenic older adults.^22^ A possible explanation for these contradictory results is that our study has focused on primary sarcopenia, while in most previous CAF investigations, sarcopenic individuals presented with other diseases, such as chronic heart failure, chronic obstructive pulmonary disease and hip fracture.^22^ Furthermore, we excluded individuals with renal diseases, as this could affect CAF concentration.^22^ We found no differences in the protein expression of selected downstream components of the agrin pathway. However, we observed an increased expression of Cav3 in the older cohorts compared with the Y controls. Cav3 is also localized at the NMJ,^27^ and among its functions, it may contribute to the clustering of ACh receptors.^23^ An increased Cav3 expression in skeletal muscle might suggest an active process of NMJ remodelling in these older individuals, potentially a compensatory response as observed in some neuromuscular disorders.^28^

Overall, altered Cav3 expression and increased agrin degradation suggested by elevated CAF concentration point towards NMJ structural destabilization that may result in impaired NMJ transmission with ageing. This latter observation is consistent with recent data showing that gene sets involved in NMJ regulation were not altered in sarcopenic compared with non‐sarcopenic individuals of different ethnicities,^29^ although a caveat of that study is that it used bulk RNA‐Seq and did not assess the signalling from single subsynaptic myonuclei.

### Motor unit loss, alterations in motor unit potential properties and underlying mechanisms

In agreement with the literature,^5^ we observed MU loss in the older cohorts compared with the young controls. While at first our number of MUs can seem rather low (on average, ~17 in Y and ~11/13 in older groups), this should be considered an index rather than an absolute value and is dependent on the MUNE technique employed. Indeed, in the present study, we employed the iMUNE method (an index computed as the ratio between the mean MUP area obtained by iEMG and muscle CSA)^21^ rather than the traditional MUNE (obtained instead as the ratio between the compound muscle action potential and the MUP area obtained from surface EMG),^5^ which is expected to be more sensitive and not biased by subcutaneous fat thickness.^21^ MU loss was not exacerbated in S. In contrast to our findings, a previous investigation observed fewer MUs in sarcopenic compared with non‐sarcopenic individuals in the vastus lateralis muscle.^12^ However, this study employed a definition of sarcopenia solely based on the assessment of muscle mass in the quadriceps muscle group. We postulate that the difference between our results and this study is due to sarcopenia classification, as we used the EWGSOP2 guidelines, considering both handgrip strength and combined lean mass of the upper and lower limbs.^2^

We observed alterations in motoneuron and MUP properties. While age‐related declines in motoneuron firing rate have been generally reported,^30^, ^31^ changes are variable among muscle groups, possibly due to their different roles (antigravity vs. non‐antigravity),^30^ innervation type (brainstem vs. spinal), amplitude and duration of afterhyperpolarization and length and diameter of the axons.^31^ In our study, the mean firing rate assessed in the vastus lateralis was reduced only in S compared with Y. This finding could be explained by motoneuron structural alterations,^1^ as supported by the observed age‐related increased neurofilament light chain concentration, a circulating biomarker of axonal damage,^9^ although not specific to motoneurons. Reduced persistent inward current, regulating motoneuron excitability, may also play a role,^32^ but its contribution to diagnosed sarcopenia has yet to be determined. Reduced synaptic input onto the MU might contribute to lower mean firing rates in females but not males, as sex differences were noted in the activation capacity assessed by the interpolated twitch technique (see *Data S1*). However, S did not exhibit significantly lower mean firing rates compared with NS and PS; thus, this mechanism is unlikely to explain their greater muscle weakness, which is instead probably largely due to their smaller muscle mass. In fact, normalizing handgrip and knee extensor strength for muscle/lean mass eliminated the differences among the older groups (*Figure* *S1*).

In addition, our results showed some age‐related changes in MUP turns, NF MUP duration and NF count, overall suggesting an MUP and NF MUP shape of increased complexity. Contrary to our hypothesis, this electrophysiological profile was mostly worsened in NS. This was not driven by increased fibre diameter variability, as we observed no changes in this parameter when comparing the three old groups. Recurring denervation and reinnervation cycles could also explain changes in MUP and NF MUP complexity.^18^ Interestingly, the increase in the percentage of NCAM‐positive fibres was more pronounced in NS (3.8‐fold vs. Y), similar to our electrophysiological data. NCAM is an accepted muscle biomarker of denervation, reported to be re‐expressed in aged muscle fibres in different human investigations.^24^ More recently, it has been shown that NCAM is also re‐expressed in conditions of short‐term muscle disuse (i.e., 10‐day bed rest or unilateral lower limb suspension),^15^, ^16^ suggesting that this biomarker is also sensitive to the initial stages of neuromuscular destabilization and altered innervation pattern. This is also supported by the fact that NCAM staining is also present in large and round fibres^24^ and that its re‐expression plays a role in guiding axon reinnervation, creating a large area of attraction that promotes synaptogenesis and improves the connectivity of the existing NMJs.^33^ It is thus tempting to speculate that in NS, a more acute and dynamic state of neuromuscular remodelling is captured by our electrophysiological and molecular assessments. In PS and S, denervation processes may have become more chronic and characterized by a lower reinnervation potential,^4^, ^12^ leading to a more limited increase in MUP complexity and NCAM expression. In summary, we find that contrary to our hypothesis, although NS, PS and S differ significantly in several functional measures of mass and force, they do not differ significantly in their neuromuscular connectivity.

### Perspectives and methodological considerations

#### Considerations regarding sarcopenia definition and other confounding factors

Our findings show that neuromuscular alterations (MU loss, NMJ instability, impaired NMJ transmission, myofibre denervation and axonal damage) are present in both S and NS individuals, in which sarcopenia was diagnosed according to the EWGSOP2 definition, which considers sarcopenia as the coexistence of a condition of low muscle strength and mass.^2^ However, several studies expressed concerns regarding this definition, considering it too strict and leading to a risk of underdiagnosis.^3^, ^34^ To solve this issue, in this study, we employed the EWGSOP2 definition with a threshold modification for handgrip strength recently proposed by Westbury and colleagues, leading to higher prevalence rates of sarcopenia while preserving the capacity to predict key health outcomes.^14^ Despite the rigorous approach employed, we acknowledge that different sarcopenia definitions could lead to different results. One important strength of our study is that our older participants did not present with any major comorbidities; thus, our findings are related only to age‐related alterations and are not confounded by the impact of other diseases.

Second, volunteers' physical activity levels could have influenced our results. According to the administered physical activity questionnaire (the GPAQ), older groups were inactive compared with Y, and this is likely to mediate at least in part their impaired neuromuscular function and integrity. Indeed, periods of muscle disuse are known to impact the neuromuscular system,^15^, ^16^, ^35^ although evidence in milder models mimicking better a sedentary lifestyle is still not available.^36^ Moreover, as S was not less physically active than NS and PS, this could partially explain the absence of differences in the parameters regarding MU and NMJ degeneration.

Third, contractions up to 25% MVC were investigated; thus, we acknowledge that our iEMG findings (including iMUNE) may be limited mainly to lower threshold and slower type MUs, generally considered more atrophy resistant to neuromuscular ageing.^1^ However, glycogen depletion experiments^37^ showed that a switch from slow to fast twitch fibres occurs at ∼20% MVC in the vastus lateralis. In addition, the choice of these contraction intensities (10% and 25% MVC) allows a direct comparison with previous iEMG studies, as this limitation represents a technical constraint of iEMG (the decomposition accuracy decreases with higher contraction intensities due to MUP superimposition) and is shared with the previous literature on the topic. Alternative techniques allowing decomposition at higher contraction intensities, such as high‐density electromyography, do not enable the specific investigation of NMJ‐related parameters and are affected in their accuracy by subcutaneous fat thickness, making their application in the context of ageing challenging, particularly in females.

#### Do motor unit loss and neuromuscular junction degeneration precede sarcopenia?

Our results might suggest that MU loss and NMJ degeneration precede clinically diagnosed sarcopenia, as they can already be detected in non‐sarcopenic older adults. This concept is supported by investigations in rodents, showing that morphological signs of NMJ denervation,^38^ alterations in NMJ transcriptional and proteomic profiling^39^ and impaired recruitment of MUs and NMJ transmission^40^ were already evident before the overt loss of muscle mass and/or function with ageing. A plausible determinant of sarcopenia incidence may be the exposure time to these neurodegenerative processes, potentially due to an earlier onset in individuals who will develop sarcopenia. While this finding has never been proven in humans so far, our study suggests the need for early preventive measures. Considering the possible neuroprotective effect of regular exercise on MUs and in preventing age‐related denervation (although evidence is not always univocal),^41^ it seems likely that long‐term physical activity practice may help to slow down the progression of sarcopenia. Also, the recent discovery that regeneration of neuromuscular synapses occurs after inhibition of the gerozyme 5‐hydroxyprostaglandin^42^ may offer a new pharmacological strategy to combat sarcopenia. The electrophysiological and molecular parameters assessed in this study, NF segment jitter, iMUNE, CAF and neurofilament light chain concentration and NCAM+ myofibers, may serve as useful biomarkers of neuromuscular impairment with ageing. We acknowledge that the cross‐sectional nature of this study represents a limitation, and future longitudinal investigations are warranted to confirm these observations.

## Conclusions

This study shows that neuromuscular alterations (MU loss, NMJ instability, impaired NMJ transmission, myofibre denervation and axonal damage) are present in non‐sarcopenic, pre‐sarcopenic and sarcopenic individuals aged >70 years without major comorbidities. This finding suggests that neuromuscular alterations may precede the onset of sarcopenia, as clinically defined. There is a need to combat these neuromuscular maladaptations through exercise, nutritional and pharmaceutical approaches in order to prevent the onset and progression of sarcopenia.

## Funding information

The present work was funded by the PRIN project ‘NeuAge’ (2017CBF8NJ_001) to MVN and MAP, the Donald E. and Delia B. Baxter Foundation, and the Li Ka Shing Foundation to HMB. EM was supported by the Stanford Wu Tsai Human Performance Institute Fellowship. This work was funded by the European Union via the Horizon 2022 Research and Innovation Programme under the Marie Sklodowska‐Curie grant agreement no. 101109133 to EM. We also acknowledge co‐funding from Next Generation EU to MVN in the context of the National Recovery and Resilience Plan, Investment PE8—Project Age‐It: ‘Ageing Well in an Ageing Society’. This resource was co‐financed by the Next Generation EU (DM 1557 11.10.2022). The views and opinions expressed are only those of the authors and do not necessarily reflect those of the European Union or the European Commission. Neither the European Union nor the European Commission can be held responsible for them. [Correction added on 30 September 2024, after first online publication: The funding information has been updated in this version.]

## Conflict of interest statement

The authors have no conflict of interest to declare.

[Correction added on 30 September 2024, after first online publication: Reference 35 has been updated in this version.]

## Supporting information


**Table S1.** Details of the generalised linear mixed effect models performed for each iEMG variable. The overall estimate and P value of the model are reported in this table, while P values of the time‐point comparison are presented in the text. Motor unit potentials (MUPs) from 4160 (32.25 (13.8) on average per participant) and 6340 MUPs (49.15 (14.24) on average), sampled at 10% and 25% MVC, respectively, were analysed. Near fibre MUPs from 1724 and 2644 MUPs, sampled at 10% (13.36 (8.62) on average) and 25% (20.5 (10.05) on average) MVC, respectively, were analysed.
**Figure S1.** Knee extensors and handgrip specific force across different stages of human sarcopenia. Statistical analysis was performed using and two‐way ANOVAs. Results are shown as mean and standard deviation. Knee extensors maximum voluntary isometric force (MVC) normalised for the mean quadriceps cross‐sectional area (CSA; mean of the values at 30%, 50% and 70% of femur length) (A); Handgrip strength normalised for arm lean mass (B). Y: young individuals; NS: non‐sarcopenic; PS: pre‐sarcopenic; S: sarcopenic. **P < 0.01; ***P < 0.001.
**Figure S2.** Proteins levels of different acetylcholine receptors (AChR) subunits across different stages of human sarcopenia. Statistical analysis was performed using Kruskal‐Wallis tests. Results are shown as mean and standard deviation. AChR δ subunit (A); AChR ε subunit (B); AChR γ subunit (C). The intensity of immunostained bands was normalized to the total protein amount measured from the same membrane stained with Ponceau S Staining. Y: young individuals; NS: non‐sarcopenic; PS: pre‐sarcopenic; S: sarcopenic. Data presented for 11 Y, 21 NS, 19 PS and 8 S. **P < 0.01; ***P < 0.001.
**Figure S3.** Representative Western Blot of Muscle‐Specific Kinase total (MuSK), phosphorylated MuSK (pMuSK_Tyr755_), caveolin 3 (Cav3), docking protein 7 (Dok7), low‐density lipoprotein receptor‐related protein 4 (Lrp4) and acetylcholine receptors (AChR) subunits δ, ε and γ. Total protein amount stained with Ponceau S is reported from the same membrane of each immunostained protein. Y: young individuals; NS: non‐sarcopenic; PS: pre‐sarcopenic; S: sarcopenic.
