# Supplementary material for: Neuromuscular impairment at different stages of human sarcopenia
Source: J Cachexia Sarcopenia Muscle. 2024 Sep 5;15(5):1797–810. doi: 10.1002/jcsm.13531 (PMC11446718; doi:10.1002/jcsm.13531)
Supplement: Supplementary file 1 — Table S1. Details of the generalised linear mixed effect models performed for each iEMG variable. The overall estimate and P value of the model are reported in this table, while P values of the time‐point comparison are presented in the text. Motor unit potentials (MUPs) from 4160 (32.25 (13.8) on average per participant) and 6340 MUPs (49.15 (14.24) on average), sampled at 10% and 25% MVC, respectively, were analysed. Near fibre MUPs from 1724 and 2644 MUPs, sampled at 10% (13.36 (8.62) on average) and 25% (20.5 (10.05) on average) MVC, respectively, were analysed. Figure S1. Knee extensors and handgrip specific force across different stages of human sarcopenia. Statistical analysis was performed using and two‐way ANOVAs. Results are shown as mean and standard deviation. Knee extensors maximum voluntary isometric force (MVC) normalised for the mean quadriceps cross‐sectional area (CSA; mean of the values at 30%, 50% and 70% of femur length) (A); Handgrip strength normalised for arm lean mass (B). Y: young individuals; NS: non‐sarcopenic; PS: pre‐sarcopenic; S: sarcopenic. **P < 0.01; ***P < 0.001. Figure S2. Proteins levels of different acetylcholine receptors (AChR) subunits across different stages of human sarcopenia. Statistical analysis was performed using Kruskal‐Wallis tests. Results are shown as mean and standard deviation. AChR δ subunit (A); AChR ε subunit (B); AChR γ subunit (C). The intensity of immunostained bands was normalized to the total protein amount measured from the same membrane stained with Ponceau S Staining. Y: young individuals; NS: non‐sarcopenic; PS: pre‐sarcopenic; S: sarcopenic. Data presented for 11 Y, 21 NS, 19 PS and 8 S. **P < 0.01; ***P < 0.001. Figure S3. Representative Western Blot of Muscle‐Specific Kinase total (MuSK), phosphorylated MuSK (pMuSKTyr755), caveolin 3 (Cav3), docking protein 7 (Dok7), low‐density lipoprotein receptor‐related protein 4 (Lrp4) and acetylcholine receptors (AChR) subunits δ, ε and γ. Total protein amou [file JCSM-15-1797-s001.docx]

**Data S1: Supplementary Materials**

**Supplementary methods**

*Handgrip strength*

The measurement was obtained with the participants sitting in a chair with their elbows close to the trunk and flexed at 90°, their forearms resting on the arms of the chair and their wrists in a neutral position with their thumbs facing upward. The measure was collected three times for both hands and the highest value was used for the analysis. A normalised index was also obtained by dividing this value by the lean mass of the respective arm assessed by DEXA.

*Short Physical Performance Battery (SPPB)*

Each component was graded on a scale of 0 (worst performance, inability to complete the test) to 4 (best performance). To evaluate balance, participants were asked to stand with their feet together, followed by semi-tandem and tandem positions for 10 seconds each. Gait speed was assessed through a 4-meter walk at the participant's habitual pace, with time recorded. Lastly, participants were instructed to stand up and sit down from a chair five times as quickly as possible, with their arms folded across their chests. A standard 46 cm chair was employed. The back of the chair was secured against a wall.

*Muscle size measurements*

A 47 mm, 7.5MHz linear array transducer was employed to collect all the ultrasound images. Transmission gel was generously applied to improve the acoustic contact for all images. Two CSA scans and three longitudinal scans were obtained at each site, and the image with the best quality was selected for the analysis. Femur length was measured as the distance between the greater trochanter and the mid-patellar point, with 0% representing the mid-patellar point (distal part) and 100% the greater trochanter (proximal part). To obtain the CSA images, the transducer was moved slowly in a transverse plane from the medial border of the vastus medialis to the lateral borders of the vastus lateralis, while maintaining consistent pressure on the skin. Lf was obtained with the manual linear extrapolation method using the segmented line tool of ImageJ (1.52v; National Institutes of Health, Bethesda, MD, USA). Briefly, the visible part of the fascicle was marked and then extrapolated with a straight line until the extension of the superficial aponeurosis. PA was defined as the angle of intersection between the fascicles and the deep aponeurosis.

*Quadriceps force, rapid force production and activation capacity*

During the MVC evaluation, participants were asked to push as strongly and as fast as they could for about 4s. They received visual feedback and verbal encouragement. The three trials were separated by a 1-minute rest. The force signal was recorded at 1000 Hz using LabChart software (v.8.13, ADInstrument, Dunedin, NewZealand). During the MVC testing, the participants received electrical stimulations via a stimulator device (Digitimer DS7AH), which were applied through two pads placed on the thigh, one proximally and the other distally. One doublet stimulation was applied when the contraction reached a plateau, while the second pulse was applied one second after the contraction. The intensity of the electrical current required for each participant's supra-maximal stimulation was determined before the testing by monitoring the force output during a series of stimuli with increasing current. The current at which no further increase in force output was observed was recorded and used during the assessment. The muscle activation was calculated using the following equation:

$$Activation capacity=(1-\frac{A}{B}) x 100$$

where A represents the superimposed twitch torque and B represents the resting control twitch torque.

All the analyses were performed with a custom Python (v.3.9) script.

*Intramuscular electromyography*

The iEMG signal was recorded at 40 kHz with the LabChart software (v.8.13, ADInstruments). Visual feedback was provided during the contractions to allow participants to achieve the target constant force (10% or 25% MVC) for the required 20 seconds each. 30 s of rest was provided between contractions. The needle position was adjusted between contractions through a combination of twisting it by 180° or extracting it by 2-3 mm, so that motor units were sampled from at least three different muscle depths. The needle was carefully maintained in a constant position during recordings.

MUP trains with fewer than 35 MUPs or MUPs with signal-to-noise ratios <15 and/or non-physiological shapes were excluded. The motor unit discharge pattern was considered only for MUP trains with inter-discharge intervals having a Gaussian distribution. Regarding NF MUP analysis, only trains with signal-to-noise ratios >15, >34 NF MUPs and with a NF count >1 were included.

*Circulating biomarkers assessment*

*CAF*

Serum CAF concentration was obtained using a commercially available enzyme-linked immunosorbent assay (ELISA) kit (Human Agrin SimpleStep ELISA, ab216945, Abcam, Cambridge, UK) following the manufacturer’s instructions. Samples were diluted 1:4 and run in duplicate.

*Neurofilament light chain*

The neurofilament light chain analysis was carried out at the facility ‘Centro Piattaforme Tecnologiche’ of the University of Verona (Verona, Italy) employing the single molecule array (SIMOA) Bead Technology (Quanterix Corporation 900 Middlesex Turnpike, Billerica, MA 10821) on a Quanterix SR-x (#1913QP0444) platform with Simoa® Nf-light Advantage Kit (SR-x). Samples were diluted 1:4 and analysed in duplicate.

*BDNF*

Serum BDNF concentration was obtained using a commercially available enzyme-linked immunosorbent assay (ELISA) kit (Brain-derived neurotrophic factor ELISA, ab212166, Abcam, Cambridge, UK) following the manufacturer’s instructions. Samples were diluted 1:20 and run in duplicate.

*NT-4*

Serum NT-4 concentration was obtained using a commercially available enzyme-linked immunosorbent assay (ELISA) kit (Glial Derived Neurotrophic Factor ELISA, ELH-NT4, Raybiotech, US) following the manufacturer’s instructions. Samples were diluted 1:1 and run in duplicate.

*IL-6*

Serum IL-6 concentration was obtained using a commercially available enzyme-linked immunosorbent assay (ELISA) kit (Interleukin-6 ELISA, ab178013, Abcam, Cambridge, UK) following the manufacturer’s instructions. Samples were diluted 1:1 and run in duplicate.

*Muscle Biopsy*

The biopsy part for Western Blot analysis was frozen in liquid nitrogen and stored at −80°C, while the part for immunohistochemical analysis was included in optimal cutting temperature (OCT) compound, frozen in liquid nitrogen pre-chilled isopentane and stored at −80°C. Cryosections were cut with a manual cryostat (Leica CM3050 S), producing 10 μm-thick sections.

*Immunofluorescence analyses*

Detection and quantification of denervated myofibres were performed by NCAM immunofluorescent staining as described: serial cryosections were fixed in 4% PFA for 5 min, washed in PBS, permeabilised in 1% Triton X-100 in PBS (PBS-T) and then blocked in 5% Goat Serum + 1% BSA in PBS-T. The same cryosections were then labelled (overnight, 4°C) using mouse IgG1 antibody directed against human CD56/NCAM (clone HCD56, BioLegend, cat. 318302) and rabbit antibody directed against laminin (Abcam, cat. ab11575) 1:200 and 1:500 diluted, respectively, in 5% GS + 1% BSA in PBS-T. Sections were rinsed in PBS-T (3x5 min) and then incubated with Cy3-affinity pure goat anti-mouse IgG1 and Alexa Fluor 488 affinity pure goat anti-rabbit (Jackson Immunoresearch, cat. 115-165-205 and 111-546-045, respectively) 1:200 and Hoechst (Thermo Scientific, cat. H3570) 1:1000 diluted in 5% GS + 1% BSA in PBS-T, for1 hour at room temperature. Sections were washed in PBS-T (3x5 min) and incubated for 1 minute in 1:20 True Black Lipofuscin Autofluorescence Quencher (Biotium, cat. 23007) diluted in 70% ethanol. After washing in PBS, sections were coverslipped. Negative controls were performed by omitting the primary antibodies from sample incubations. All sections were imaged using a Keyence fluorescent microscope (Keyence, BZ-X810) at 20x magnification. Merged images were generated through a semi-automated algorithm to reconstruct the whole tissue section image. NCAM-positive fibres were counted on captured and merged images, using ImageJ software (1.52v; National Institutes of Health, Bethesda, MD) and expressed as the number of positive myofibres per total number of myofibres detected in the biopsy area by laminin staining (400-2000 muscle fibres). The variability of muscle fibres diameters was expressed as the coefficient of variation (CV; standard deviation/mean of all the muscle fibres visible on the biopsy section).

*Western Blot Analysis*

Frozen muscle samples were pulverized and immediately re-suspended in a lysis buffer (20 mM Tris-HCl, 1% Triton X100, 10% Glycerol, 150 mM NaCl, 5 mM EDTA, 100 mM NaF and 2 mM NaPPi supplemented with 5× Protease Inhibitor (Protease Inhibitor Cocktail, Sigma-Aldrich, St. Louis MO), 1× phosphatase inhibitors (Phosphatase Inhibitor Cocktail, Sigma-Aldrich) and 1mM PMSF. The homogenate obtained was incubated on ice for 40 min and then centrifuged at 18000 × g for 20 min at 4°C. Total protein concentration was determined for each sample using an RC DC™ Protein Assay kit (Bio-Rad Laboratories, Inc., Hercules, CA, USA). Equal amounts of muscle samples (40 μg) were denatured and separated on 4-20% gradient precast gels (Bio-Rad Laboratories, Inc.). Due to the elevated number of samples under investigation, a reference sample was prepared and loaded in every gel during the same experimental session to make the comparison possible among the samples. After the gel run, proteins were electro-transferred to PVDF membranes at 35 mA overnight. The membranes were blocked using 5% nonfat dry milk in TBST (0.02 M Tris, 0.05 M NaCl, 0.1% Tween-20) for 2 hours at room temperature, rinsed with TBST and subsequently probed with specific primary antibodies (see below), in blocking solution overnight at 4°C. Thereafter, the membranes were incubated for 1 hour at room temperature with corresponding horseradish peroxidase (HRP)-conjugated secondary antibodies. Protein bands were visualized by an enhanced chemiluminescence method (Amersham ECL Select™, GE Healthcare, Little Chalfont, UK). The content of each protein investigated was assessed by determining the Brightness–Area Product of the protein band normalized to total protein content, obtained by Ponceau S staining (0.2% Ponceau Red in 3% acetic acid) or evaluated between phosphorylated and unphosphorylated total forms of the same protein (expressed as arbitrary units, AU). Antibodies used were: anti-mouse AChRδ (1:1000, MA3-043; Thermo Fisher Scientific, Waltham, MA, US), anti-rabbit AChRε (1:1000, PA5-87600; Thermo Fisher Scientific), anti-rabbit AChRγ (1:1000, PA5-103556; Thermo Fisher Scientific), anti-rabbit Cav3 (1:1000, GTX109650; GeneTex Inc., Irvine, CA, US), anti-rabbit Dok7 (1:1000, ab75049; Abcam, Cambridge, UK), anti-rabbit Lrp4 (1:1000, ab230188; Abcam), anti-rabbit MuSK (1:1000, ab92950; Abcam), anti-rabbit *p*MuSK_Tyr755_ (1:1000, ab192583; Abcam); rabbit anti-mouse IgG (1:5000, P0161; Dako North America Inc., Carpinteria, CA, US); goat anti-rabbit IgG (1:10000, #7074; Cell Signalling Technology, Inc., Danvers, MA, USA).

*Statistical analysis*

The following is the list of variables not normally distributed, in which non-parametric statistics was applied: CAF, neurofilament light chain and IL-6 concentration, and the biomarkers derived from muscle biopsies (NCAM, variability of muscle fibres diameter and all the proteins assessed from the Agrin-Lrp4-MuSK-Dok7 pathway). Non-parametric statistics was also employed for GPAQ, SPPB and balance scores, representing categorical variables. As explained in the main text, iEMG data were analysed using generalised linear mixed models (fixed effects: group and sex; cluster variable: subject). The family of distribution used in the analysis varied depending on each variable, with the gamma or inverse Gaussian distribution being employed. Different link functions were associated with each distribution (Table S1). The Bayesian information criterion (BIC) was used to compare the models. When multiple models had similar minimal BIC values, the canonical link function for the respective distribution was selected (i.e. inverse function (1/y) for gamma distribution and inverse squared (1/y^2^) for inverse Gaussian distribution). Post hoc comparisons were carried out using the Holm correction.

**Supplementary results**

*Sex differences in muscle function and physical performance*

Handgrip strength (P < 0.0001, ηp^2^ = 0.65) and MVC showed a large effect of sex (P < 0.0001; ηp^2^ = 0.41), with females (F) exhibiting lower isometric forces than males (M). However, no group*sex interaction was found for both variables. The knee extensors specific force (MVC/CSA) was slightly superior in M (P = 0.0114; ηp^2^ = 0.05), with no interaction. No sex differences were observed for handgrip strength normalised for arm lean mass. TTP63% did not display any effect of sex or interaction. Activation capacity showed a small effect of sex (P = 0.0432; ηp^2^ = 0.03) and a significant interaction (P = 0.0423; ηp^2^ = 0.07). Indeed, M have slightly higher activation capacity values; while in F, differently to M, the comparison between Y and S was significant (P = 0.0161). No significant effect of sex was found for SPPB and its components: CST time, balance score and gait speed.

*Sex differences in muscle morphology*

All DEXA parameters showed an effect of sex: ALM (P < 0.0001; ηp^2^ = 0.72), ALM/h^2^ (P < 0.0001; ηp^2^ = 0.51), leg lean mass (P < 0.0001; ηp^2^ = 0.69), with F presenting lower lean mass values. These were observed without significant group*sex interaction. Quadriceps and vastus lateralis CSA_mean_ presented a large effect of sex (quadriceps: P < 0.0001; ηp^2^ = 0.56; vastus lateralis: P < 0.0001; ηp^2^ = 0.41) and a significant interaction (quadriceps: P = 0.0099; ηp^2^ = 0.09; vastus lateralis: P = 0.0103; ηp^2^ = 0.09). Specifically, F presented a smaller CSA_mean_. Moreover, for VL in F, no differences were found in S compared to NS and PS, differently from M (NS vs S: 0.0096; PS vs S: 0.0357). Regarding muscle architecture parameters, Lf showed no group*sex interaction but an effect of sex (P = 0.0006; ηp^2^ = 0.09) was observed, pointing toward longer fascicles in M. Similar trend was observed for PA, with larger angles observed in M (P = 0.0105; ηp^2^ = 0.05).

*Sex differences in iMUNE, MUP properties and NMJ transmission*

The iMUNE showed an effect of sex (P < 0.0001; ηp^2^ = 0.14), indicating higher values in M, with no group*sex interaction. No effect of sex and interaction were detected in all the iEMG parameters considered. Thus, this variable was excluded from the final generalised linear mixed models presented in the main manuscript.

*Sex differences in circulating biomarkers of neurodegeneration*

No effect of sex was noticed for CAF, neurofilament light chain and IL-6 concentration. Similarly, no significant effect of sex and group*sex interaction was observed for BDNF and NT-4.

*Sex differences in muscle biomarkers of NMJ instability and denervation*

Due to the more limited sample size, as muscle biopsies were collected only in a subgroup of participants, sex differences were not investigated for muscle biomarkers of NMJ instability and denervation.

**Supplementary tables and figures**

| Parameter | Distribution | Link function | Estimate | 95% CI | P value  Group |
| --- | --- | --- | --- | --- | --- |
| MUP area 10%  (µV · ms) | Inverse gaussian | Identity | 950 | 915.21 to 985.1 | 0.075 |
| MUP area 25%  (µV · ms) | Inverse gaussian | Identity | 1245.448 | 1208.3 to 1282.64 | 0.1674 |
| MUP duration 10%  (ms) | Gamma | Inverse | 0.0908 | 0.087 to 0.0946 | 0.8784 |
| MUP duration 25%  (ms) | Gamma | Inverse | 0.0868 | 0.0834 to 0.0901 | 0.4675 |
| MUP turns 10%  (number of turns) | Inverse gaussian | Inverse squared | 0.1025 | 0.0945 to 0.1104 | ***0.0798*** |
| MUP turns 25%  (number of turns) | Inverse gaussian | Inverse squared | 0.1037 | 0.0965 to 0.1108 | ***0.0345*** |
| Mean firing Rate 10%  (Hz) | Inverse gaussian | Identity | 8.954 | 8.65 to 9.2557 | 0.2694 |
| Mean firing Rate 25%  (Hz) | Inverse gaussian | Identity | 9.782 | 9.47 to 10.0955 | ***0.0091*** |
| NF MUP duration 10%  (ms) | Gamma | Inverse | 0.3425 | 0.3223 to 0.3627 | ***0.0093*** |
| NF MUP duration 25%  (ms) | Gamma | Inverse | 0.3652 | 0.3462 to 0.3842 | ***0.0037*** |
| NF count 10%  (number) | Inverse gaussian | Inverse | 0.766 | 0.733 to 0.799 | ***0.0027*** |
| NF count 25%  (number) | Inverse gaussian | Inverse | 0.8099 | 0.782 to 0.8376 | ***0.0008*** |
| NF MUP segment  jitter 10% (µs) | Gamma | Inverse | 0.0255 | 0.0245 to 0.0246 | 0.1905 |
| NF MUP segment  jitter 25% (µs) | Gamma | Inverse | 0.0216 | 0.0214 to 0.0219 | ***<0.0001*** |

**Table S1:** Details of the generalised linear mixed effect models performed for each iEMG variable. The overall estimate and P value of the model are reported in this table, while P values of the time-point comparison are presented in the text. Motor unit potentials (MUPs) from 4160 (32.25 (13.8) on average per participant) and 6340 MUPs (49.15 (14.24) on average), sampled at 10% and 25% MVC, respectively, were analysed. Near fibre MUPs from 1724 and 2644 MUPs, sampled at 10% (13.36 (8.62) on average) and 25% (20.5 (10.05) on average) MVC, respectively, were analysed.


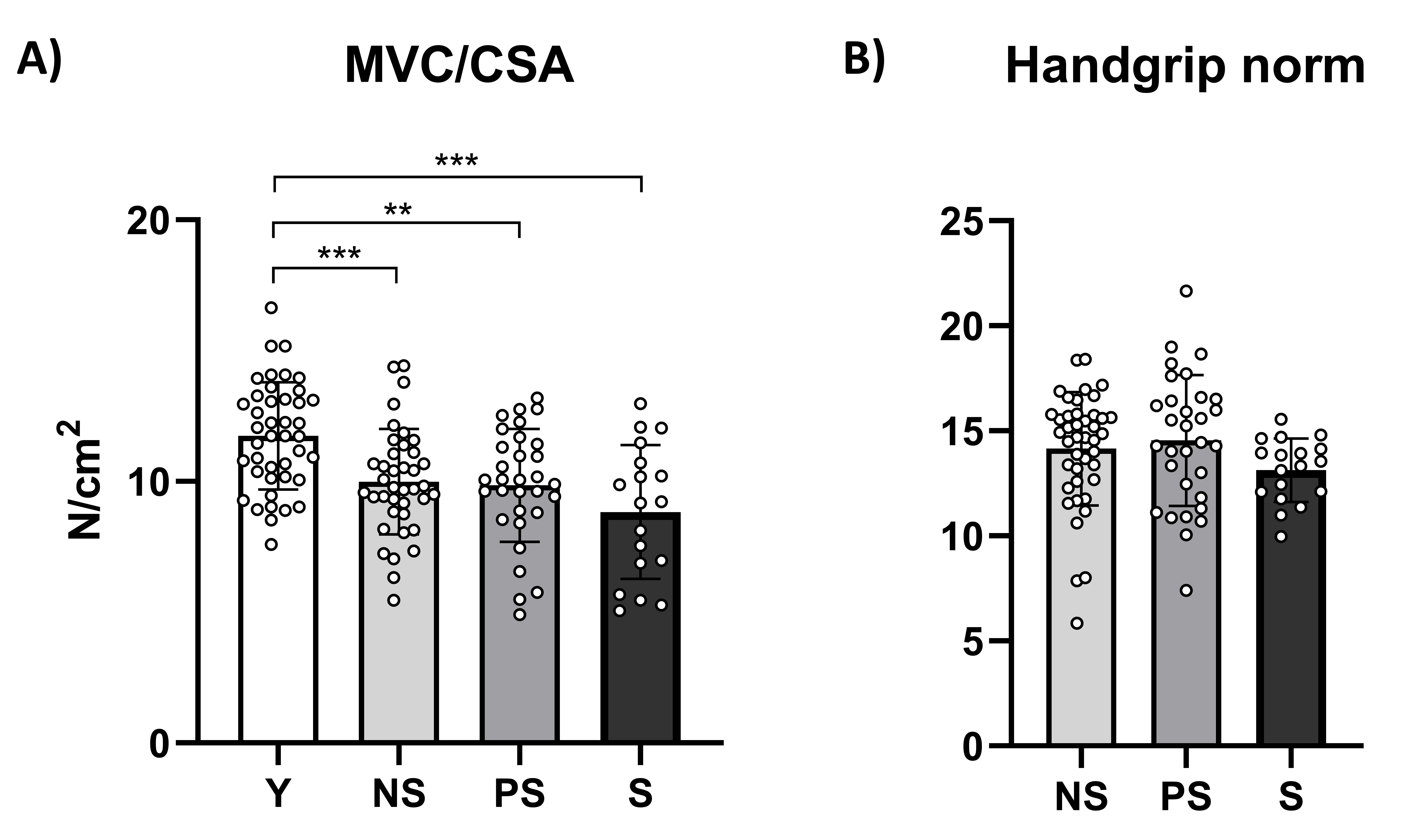


**Figure S1:** Knee extensors and handgrip specific force across different stages of human sarcopenia. Statistical analysis was performed using and two-way ANOVAs. Results are shown as mean and standard deviation. Knee extensors maximum voluntary isometric force (MVC) normalised for the mean quadriceps cross-sectional area (CSA; mean of the values at 30%, 50% and 70% of femur length) (A); Handgrip strength normalised for arm lean mass (B). Y: young individuals; NS: non-sarcopenic; PS: pre-sarcopenic; S: sarcopenic. **P < 0.01; ***P < 0.001


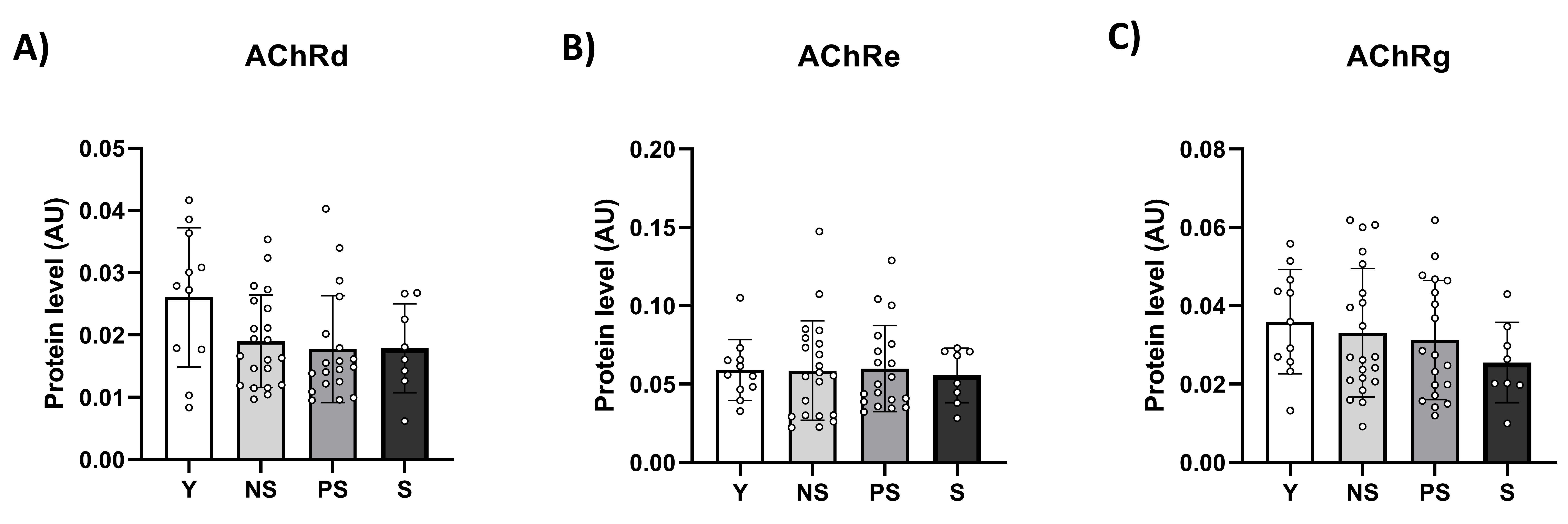


**Figure S2:** Proteins levels of different acetylcholine receptors (AChR) subunits across different stages of human sarcopenia. Statistical analysis was performed using Kruskal-Wallis tests. Results are shown as mean and standard deviation. AChR δ subunit (A); AChR ε subunit (B); AChR γ subunit (C). The intensity of immunostained bands was normalized to the total protein amount measured from the same membrane stained with Ponceau S Staining. Y: young individuals; NS: non-sarcopenic; PS: pre-sarcopenic; S: sarcopenic. Data presented for 11 Y, 21 NS, 19 PS and 8 S. **P < 0.01; ***P < 0.001

**
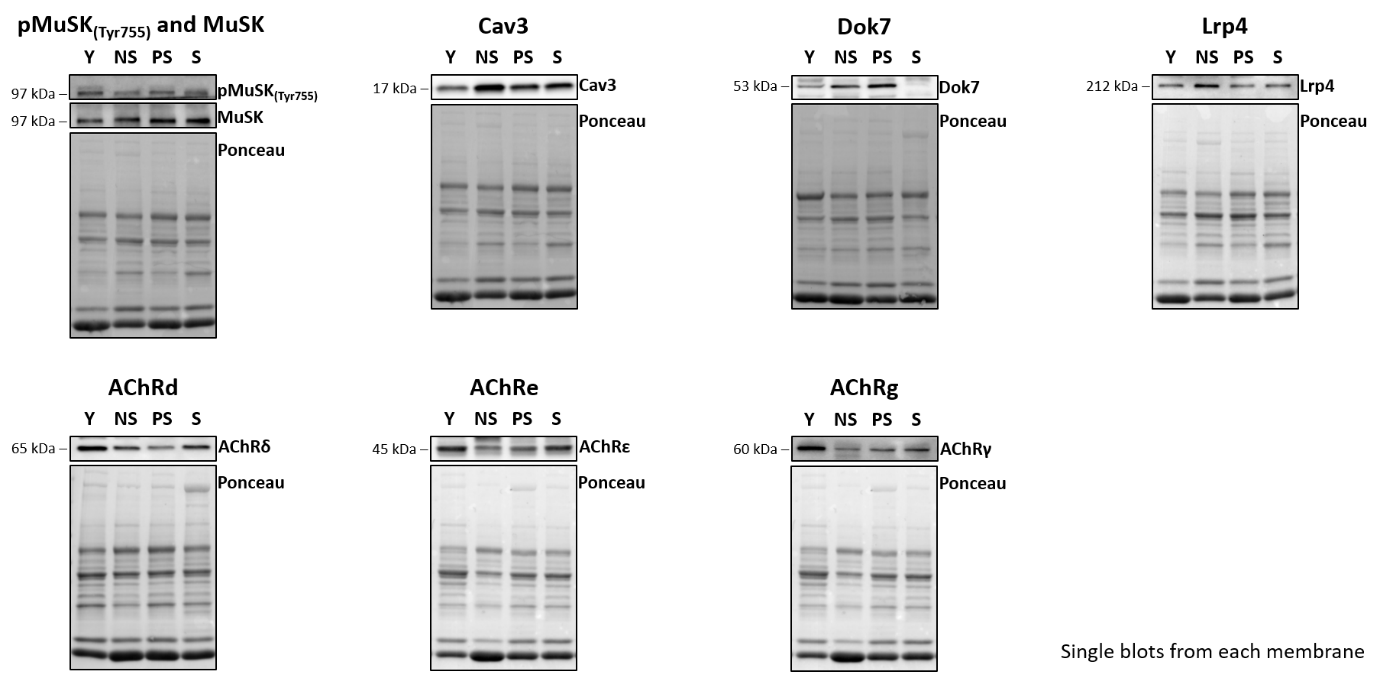
**

**Figure S3:** Representative Western Blot of Muscle-Specific Kinase total (MuSK), phosphorylated MuSK (pMuSK_Tyr755_), caveolin 3 (Cav3), docking protein 7 (Dok7), low-density lipoprotein receptor-related protein 4 (Lrp4) and acetylcholine receptors (AChR) subunits δ, ε and γ. Total protein amount stained with Ponceau S is reported from the same membrane of each immunostained protein. Y: young individuals; NS: non-sarcopenic; PS: pre-sarcopenic; S: sarcopenic.
